# Tomato and Melon *Meloidogyne* Resistant Rootstocks Improve Crop Yield but Melon Fruit Quality Is Influenced by the Cropping Season

**DOI:** 10.3389/fpls.2020.560024

**Published:** 2020-11-05

**Authors:** Alejandro Expósito, Montserrat Pujolà, Isabel Achaerandio, Ariadna Giné, Nuria Escudero, Aïda Magdalena Fullana, Marina Cunquero, Pablo Loza-Alvarez, F. Javier Sorribas

**Affiliations:** ^1^Department of Agri-Food Engineering and Biotechnology, Universitat Politècnica de Catalunya, Esteve Terradas, Castelldefels, Spain; ^2^Institut de Ciències Fotòniques (ICFO), The Barcelona Institute of Science and Technology, Castelldefels, Spain

**Keywords:** crop yield losses, *Cucumis melo*, *C. metuliferus*, plant tolerance, root-knot nematodes, *Solanum lycopersicum*

## Abstract

Four rotation sequences consisting of ungrafted tomato cv. Durinta – melon cv. Paloma or tomato grafted onto the resistant rootstock ‘Aligator’ – melon grafted onto the resistant *Cucumis metuliferus* accession BGV11135, and in reverse order, were conducted from 2015 to 2017 in a plastic greenhouse infested or not with *Meloidogyne incognita* to determine the plant tolerance (*T*), the minimum relative crop yield (*m*) and fruit quality. The relationship between *M. incognita* densities in soil at transplanting (*Pi*) of each crop and the crop yield was assessed and *T* and *m* were estimated by the Seinhorst’s damage model. In addition, the volume and the number of nuclei of single giant cells and the number of giant cells, its volume and the number of nuclei per feeding site in susceptible tomato and melon were compared to those in the resistant tomato and *C. metuliferus* 15 days after nematode inoculation in pot test. The relationship between the *Pi* and the relative crop yield fitted the Seinhorst’s damage model in both ungrafted and grafted tomato and melon, but not for all years and cropping seasons. The estimated *T* for ungrafted and grafted tomato did not differ but *m* was lower in the former (34%) than the latter (67%). Sodium concentration in fruits from ungrafted but not from grafted tomato increased with nematode densities in spring 2015 and 2016. The estimated ungrafted melon *T* did not differ from the grafted melon cultivated in spring, but it did when it was cultivated in summer. The relative crop yield of ungrafted melon was lower (2%) than the grafted cultivated in spring (62%) and summer (20%). Sodium concentration in melon fruits from ungrafted plants increased with nematode densities. No variations in fruit quality from grafted melon cultivated in spring were found, although less dry matter and soluble solid content at highest nematode densities were registered when it was cultivated in summer. Lower number of giant cells per feeding site was observed in both susceptible tomato germplasms compared to the resistant ones but they were more voluminous and held higher number of nuclei per giant cell and per feeding site.

## Introduction

Tomato (*Solanum lycopersicum*) and melon (*Cucumis melo*) are two of the major horticultural crops worldwide with annual productions of 5.163.466 and 655.677 tonnes in 2017, respectively (FAOSTAT, 2017). Root-knot nematodes (RKN), *Meloidogyne* spp., are one of the most important limiting soil borne pathogens for vegetable production (Hallmann and Meressa, 2018). Among the more than 100 RKN species described, *M. arenaria, M. incognita, M. javanica* and *M. hapla* are the most damaging species, which are worldwide distributed, have a wide range of host plants and reproduce by parthenogenesis (Jones et al., 2013), allowing an exponential increase of nematode densities at the end of the crop from low densities at planting (Greco and Di Vito, 2009).

RKN are obligate sedentary endoparasitic nematodes. The infective second-stage juvenile (J2) moves between the soil particles and penetrates the host plant roots near to the elongation zone. The J2 moves intercellularly to the root tip, turns after the casparian strips, enter into the vascular cylinder to establish a feeding site and becomes sedentary. A feeding site is composed by five to seven multinucleate and hypertrophied cells, called giant cells, which supply nutrients to the nematode for the rest of its life cycle (Abad et al., 2009). After that, the parasitic J2 undergoes three molts to reach the adult female that lays the eggs in a gelatinous matrix, the egg mass, located outside or into the root. The embryogenesis leads to the J1 that molts inside the egg and becomes J2 until hatching occurs.

The hypertrophy and hyperplasia of root parenchyma cells lead to the formation of galls that reduce the water and nutrients uptake in the infected plants, which can show aboveground symptoms, such as, dwarfism, wilting and nutrient deficiency. The severity of the symptoms can range from asymptomatic to plant death depending on nematode densities in soil and the plant tolerance. Crop yield losses due to RKN under different environmental conditions have been summarized by Greco and Di Vito (2009). Regarding fruiting vegetables cultivated under protected or open fields, maximum yield losses of 88% and 75% have been reported for ungrafted and grafted cucumber onto *Cucurbita* hybrid rootstock, respectively; 65% and 57% for ungrafted and grafted melon onto *Cucurbita* hybrid rootstock; 56% for tomato; 39% for zucchini; and 37% for watermelon (Ploeg and Phillips, 2001; Kim and Ferris, 2002; Giné et al., 2014, 2017; López-Gómez et al., 2014; Vela et al., 2014). In addition, RKN could affect fruit quality reducing its nutritive value. For instance, Vinay (2018) reported a reduction of the lycopene content in tomato fruits up to 37% and an increase of titratable acidity, total soluble solids and vitamin C up to 20%, 75% and 21% respectively, when plants were cultivated in soil inoculated at a rate of 6 J2 g^–1^ of soil compared to the non-inoculated.

RKN control has been mainly conducted by non-fumigant and fumigant nematicides (Nyczepir and Thomas, 2009). However, the current legal regulations, such as the European directive 2009/128/CE, promote the use of alternative control methods in order to reduce their harmful effects to the environment and human health. Plant resistance has been proven to be an effective, economic, environmental and human health friendly control method against RKN (Sorribas et al., 2005; Starr and Mercer, 2009; Williamson and Roberts, 2009) able to be used in integrated nematode management strategies. Plants bearing resistance genes lead to an incompatible plant-RKN interaction by the activation of several plant genes that suppress giant cell formation and/or induction of cell apoptosis affecting nematode development and/or reproduction (Shukla et al., 2018). Plant resistance genes to some RKN species have been reported in several crops (reviewed in Williamson and Roberts, 2009), but only a few of them have been introgressed into commercial fruiting vegetable cultivars including tomato and pepper. Nonetheless, several sources of plant resistance against RKN that are able to be used in plant breeding programs or as rootstocks have been reported (Lee et al., 2010). Commercial RKN resistant rootstocks are currently available for aubergine, pepper, and tomato. Regarding cucurbit crops, the watermelon rootstock *Citrullus amarus* ‘Strongback’, released by the USDA-ARS (Kemble et al., 2019), will be commercially available soon. But currently, there is none available for melon or cucumber although RKN resistant wild *Cucumis* species that could be used in breeding programs or as rootstocks have been described, such as *C. africanus, C. anguria, C. dipsaceus, C. ficifolius, C. hystrix, C. metuliferus, C. myriocarpus, C. proferatum, C. pustulatus, C. subsericeus, C. zambianus* and *C. zeyheri* (Liu et al., 2015; Expósito et al., 2018; Cheng et al., 2019). Despite the effectiveness of plant resistance against RKN, virulent nematode populations able to circumvent plant defense mechanisms can be selected after repeated cultivation of resistant plants bearing the same *R*-gene (Verdejo-Lucas et al., 2009; Thies, 2011; Ros-Ibáñez et al., 2014; Expósito et al., 2019). Consequently, plant resistance will be effective and durable only if it is adequately used, as for example in rotation sequences with different resistance genes. In a previous study, cropping melon grafted onto *C. metuliferus* followed by tomato grafted onto the resistant rootstock ‘Aligator’ or viceversa, reduced the reproduction rate of the nematode and yielded more compared to ungrafted crops; and also reduced the level of virulence to the *Mi*1.2 gene after cropping grafted melon onto *C. metuliferus* (Expósito et al., 2019).

Grafting vegetables onto resistant rootstocks is an effective management method against biotic and abiotic stresses that also provide yield stability (Rouphael et al., 2018). However, physicochemical fruit quality, storability, and nutritive value can be affected by grafting, being necessary the knowledge of particular scion-rootstock compatibility to be used by growers (Kyriacou et al., 2017). In order to know the tolerance of grafted plants to RKN, two parameters have to be considered: the tolerance limit (*T*), that is, the maximum nematode population that do not cause crop yield losses, and the minimum relative yield (*m*) at high nematode densities (Seinhorst, 1998).

Thus, the main objective of this study was to determine the plant tolerance, the minimum relative crop yield and fruit quality of ungrafted and grafted tomato cv. Durinta onto the resistant rootstock ‘Aligator’, and ungrafted and grafted melon cv. Paloma onto the resistant *C. metuliferus* accession BGV11135, cultivated in a rotation sequence of ungrafted tomato-ungrafted melon, grafted tomato-grafted melon and viceversa, conducted from 2015 to 2017 in plots infested or not with *M. incognita* in a plastic greenhouse. In addition, histopathology analyses were conducted to determine the number and the volume of giant cells per feeding site and the number of nuclei per giant cell and per feeding site in susceptible tomato and melon and being compared to those in the resistant germplasm 15 days after nematode inoculation in pot test.

## Materials and Methods

### Plant Material

The susceptible tomato cv. Durinta (Seminis Seeds, United States and Canada) (T), the resistant tomato rootstock ‘Aligator’ (previously PG76) (Gautier seeds, France) (GT), the susceptible melon cv. Paloma (Fitó Seeds, Spain) (M), and the resistant *C. metuliferus* accession BGV11135 (GM) (Institute for Conservation and Improvement of Valencian Agrodiversity collection, COMAV-UPV, Valencia, Spain) were used in the plastic greenhouse experiment conducted to determine the damage function models, and the effect of grafting and nematode densities in fruit quality parameters. Plantlets were produced by the commercial nursery HishtilGS (Malgrat de Mar, Spain). Rootstocks seeds of tomato and melon were germinated in 104-cell polystyrene trays, and those of tomato and melon cultivars in 216-cell polystyrene trays during 2 days in a growth chamber at 25°C ± 1 °C and 90% relative humidity in the darkness. After that, plantlets were transferred to a greenhouse bench. Plantlets were watered and weekly fertilized with a 5-3-7 NPK liquid fertilizer. After 15 days, melon plants were grafted using the one cotyledon grafting method (Davis et al., 2008). Tomato plants were grafted after 25 days using the tube grafting method (Lee et al., 2010). Grafted plants were placed in a healing room at 25 °C ± 1 °C and 90% relative humidity for 5 days. After that, plants were acclimated in the shadow for 1 day and then, were transferred to a greenhouse bench for 10 days before transplanting.

The optical histopathology study was conducted with the majority of plant material used in the plastic greenhouse experiment, but the resistant tomato rootstock ‘Aligator’ was replaced by the resistant tomato cv. Monika (Syngenta Crop Protection AG, Basel, Switzerland), because it was no longer commercially available in Spain at the time of the study was conducted. Seeds were sown into vermiculite and incubated at 25 °C ± 2 °C and 16:8 h light:dark photoperiod in a growth chamber. Three-leaf stage plants were transferred to 200 cm^3^ pots filled with sterilized sand at 121 °C for 1 h and repeated after 1 day. Afterward, plants were fertilized with a slow release fertilizer (15% N, 9% P_2_O_5_, 12% K_2_O, 2% MgO2, microelements: Osmocote Plus), watered as needed and maintained in a growth chamber at the same growing conditions described previously until nematode inoculation.

### Damage Function Models

The experiment was conducted over three growing seasons (2015, 2016 and 2017) in a 700 m^2^ experimental plastic greenhouse located in Viladecans (Barcelona, Spain). The plastic greenhouse management history, the characteristics of the experiment and its design are described in Expósito et al. (2019). In brief, the experiment consisted of eight treatments replicated 10 times: grafted tomato (GT), grafted melon (GM), tomato (T) and melon (M) cultivated in both *M. incognita* infested and non-infested plots. Four individual rotation schemes were conducted in the same plots in 2015 and 2016: GT-GM, T-M, GM-GT and M-T from March to July (spring crop) and July to November (summer crop). In 2017 only the spring crop was carried out. Grafted and ungrafted melon and tomato were cultivated from April to August and from April to September, respectively. Individual plots consisted in a row of 2.5 m long and 1.5 m wide containing 4 plants spaced 0.55 m between them. Plots were spaced 0.9 m within a row and 1.5 m between rows. The soil of each plot was prepared separately to avoid cross contamination. The soil was loamy sand textured, with 1.8 organic matter (w/w) and 0.5 dS m^–1^ electric conductivity. Plants were irrigated and fertilized by a drip irrigation system with a solution of NPK (15-5-30) at 31 kg ha^–1^, and iron chelate and micronutrients at 0.9 kg ha^–1^. Weeds were removed manually during the growing seasons. Soil temperature and water content were recorded with four sensors (5TM digital soil probes, Decagon Devices, Inc.) at 1 h intervals placed at a depth of 15 cm randomly in the plots. Tomato and melon fruits were collected and weighed when they reached the commercial standards, and the relative crop yield was calculated as the crop yield in a RKN infested plot in relation to the mean crop yield in non-infested plots. The nematode population densities were determined at transplanting (*Pi*) and consisted of eight cores taken from the upper 30 cm of the soil with a 2.5 cm diameter auger, mixed and sieved through a 4 mm-pore sieve to remove stones and roots. For each experimental plot, J2 were extracted from 500 cm^3^ of soil using Baermann trays (Whitehead and Hemming, 1965) and incubated at 27°C ± 2°C for 1 week. Then, the J2 were collected with a 25 μm aperture screen sieve, counted, and expressed as J2 250 cm^–3^ of soil. The relationship between *Pi* and the relative crop yield (kg plant^–1^) was estimated per each crop to determine its compliance with the Seinhorst damage function model (*y* = *m* + (1-*m*) 0.95 ^(^*^*Pi*^*^/^*^*T*^*^–1)^) (Seinhorst, 1998).

### Fruit Quality Assessment

The third tomato cluster at the red ripening stage and one melon fruit when fully slip per each plant, when they were available, were used for fruit quality analyses. Fruits were conserved at 10 °C ± 1 °C until processed. All the parameters were analyzed twice. When it was available, the official methods of analysis (AOAC) were used (George and Latimer, 2019). Tomato and melon color was determined by using a Minolta colorimeter CR-400 model (Minolta Camera, Osaka, Japan) in the CIElab color space. Lightness (*L*^∗^), *a*^∗^ and *b*^∗^ values were recorded, and hue angle (*H*) and chroma (*C*^∗^) parameters were calculated as: *H* = tan^–1^(*b*^∗^/*a*^∗^) and chroma: *C*^∗^ = (a^*2^ + b^*2^)^1/2^. Fruit flesh firmness was measured using a Texture Analyzer TA.TXPlus (Stable Microsystems, Ltd., United Kingdom) interfaced to a personal computer. Firmness was evaluated as the maximum force (*N*) needed to depress 4 mm into the fruit with a 4 mm diameter stainless steel flat end probe (P/4). Six measurements were conducted by sample for color and firmness. Chemical analyses were conducted from melon and tomato flesh obtained by crushing melon flesh from each single melon or all tomato fruits from each cluster. The soluble solid content (*SSC*) was measured with a digital refractometer (model PR-101, Atago, Co., Tokyo, Japan) at 20°C and the results were expressed as°Brix. The pH and titratable acidity (*TA*) were determined according to AOAC 981.12 and AOAC 942.15, respectively, and expressed as g citric acid ⋅ kg^–1^ dry weight (*dw*). The dry matter content was obtained following the gravimetric method (AOAC 931.04) and was expressed as percentage of the fruit dry weight in relation to the fresh fruit weight. After that, dried samples were kept in a muffle furnace and incinerated at 475°C until white ashes were obtained (AOAC 940.26). Then, mineral content was assessed. Sodium and potassium content were determined by flame atomic emission spectrometry Corning 410 C (England). Iron, calcium and magnesium were determined by atomic absorption spectrometry Varian SpectrAA-110 (Australia). The results were expressed as g kg^–1^ *dw*, except for iron (mg kg^–1^ *dw*). Ascorbic acid content was measured using a titration method (AOAC 967.21) and oxalic acid as an extracting solution (Teixeira et al., 2012) and the results were expressed in g of ascorbic acid ⋅ kg^–1^ *dw*. The total phenolic content (*TPC*) of oxalic-aqueous extract was assessed according to the Folin-Ciocalteu assay (Singleton et al., 1999) and the results were expressed as g of gallic acid equivalent (*GAE*) kg^–1^ *dw*. The antioxidant activity of the oxalic-aqueous extracts of fruit samples was performed using the oxygen radical absorbance capacity (*ORAC*) assay (Gorjanovic et al., 2013). The results were expressed as mmol of Trolox equivalents (*TE*) kg^–1^ *dw*. Carotenoid extracts were obtained as proposed by Rodriguez-Amaya and Kimura (2004). Total carotenoid content was analyzed by UV-Vis Spectrophotometry following the method stated by Scott (2001). Melon extracts were measured at λ = 450 nm (β-carotene, maximum absorbance) and tomato extracts at λ = 470 nm (lycopene, maximum absorbance) in a Nicolet Evolution 300 Spectrophotometer (Thermo electron Corporation, Basingstoke, United Kingdom). Results were expressed in mg of carotenoid kg^–1^ *dw* (β-carotene for melon; lycopene for tomato).

### Optical Histopathology

A histopathology study with laser-scanning confocal microscopy of cleared galled-roots was performed. Three-leaf stage plants of the susceptible tomato cv. Durinta and melon cv. Paloma and the resistant tomato cv. Monika and *C. metuliferus* BGV11135 were transplanted in 200 cm^3^ pots filled with sterilized sand. Five days later, 1 or 3 *M. incognita* J2 cm^–3^ of soil were added to the pots with nematode susceptible or resistant plants, respectively, into two opposite holes of 3 cm depth and 1 cm from the stem. In order to obtain the nematode inoculum, eggs were extracted from tomato roots by blender maceration in a 5% bleach solution (40 g L^–1^ NaOCl) for 5 min (Hussey and Barker, 1973). Then, the suspension was filtered through a 74 μm sieve screen to remove root debris, and eggs were collected on a 25 μm sieve screen and placed on Baermann trays (Whitehead and Hemming, 1965) maintained at room temperature. J2 emerged during the first 24 h were discarded. After that, J2 were collected on a 25 μm sieve screen every 2 days for 6 days and kept at 9 °C until inoculation. Fifteen days after the nematode inoculation, 10 galled-root pieces per each plant were taken. Galled-root pieces were fixed, clarified and stored following the procedure described in Cabrera et al. (2018) with some modifications. In brief, galled-root pieces were handpicked and introduced in a vial containing 1 mL of sodium phosphate buffer (10 mM, pH = 7). The pieces were fixed in sodium phosphate buffer (10 mM, pH = 7) with glutaraldehyde 4% under soft vacuum for 15 min, and maintained at 4°C overnight. Afterwards, pieces were rinsed for 10 min with sodium phosphate buffer and sequentially dehydrated for 20 min in 30, 50, 70 and 90% ethanol solutions, and finally in pure ethanol for 60 min. Clarification was conducted in a solution 1:1 v/v EtOH: BABB (1:2 v/v benzyl alcohol: benzyl benzoate) for 20 min, followed by 20 min in BABB solution at room temperature. The galls were then left in an automatic tube-shaker at 4°C for 2 weeks. Afterwards, the samples were stored at 4°C. The cleared galls were imaged with laser-scanning confocal microscopy. This allowed to determine: the number of nuclei and giant cells (GC) per feeding site and the volume of each GC. The thinnest galls were selected and mounted in #1.5 bottom-glass petri dishes and fully embedded in BABB solution. Fluorescence images were acquired with an inverted Leica TCS 5 STED CW microscope (Leica Microsystem) equipped with a 10 × 0.40NA HCX Pl Apo CS air objective. The different structures within the cleared galls produced different autofluorescence spectra, partly overlapping. Two different excitation-emission schemes were used to separate them. Thus, the root cell walls of the samples were excited with a 488 nm argon laser and the fluorescence emission was collected with a hybrid detector in the range of 498–550 nm. The nuclei of GC and the nematodes was visualized with 633 nm HeNe laser and the fluorescence emission was collected with a hybrid detector in the range of 643–680 nm. Depending on the sample, the visualized volume had a thickness ranging from 60 to 170 μm. Each volume was optically sectioned to produce a collection of Z-stack images (step size of 2–3 μm). Representative frames of each crop variety are shown in detail in Supplementary Figure S1 and Supplementary Video S1. A three-dimensional (3D) reconstruction of the full imaged volume of susceptible melon cv. Paloma is shown in Supplementary Video S2. For the GC volume measurements, images were segmented using TrakEM2 ImageJ plugin (ImageJ, version 1.50i). The 3D gall reconstructions were done with Huygens software (Huygens SVI, Netherlands).

### Statistical Analyses

Statistical analyses were performed using the SAS system V9 (SAS Institute, Inc., Cary, NC, United States). The non-linear procedure proc nlin was used to determine the compliance of the relationship between the initial population densities (*Pi*) and the relative crop yield (*y*) with the Seinhorst damage-function model *y* = *m* + (1-*m*) 0.95 ^(^*^*Pi/T–*^*^1)^ when *Pi* ≥ *T*, and *y* = 1 when *Pi* < *T*, where *m* is the minimum relative yield, and *T* is the tolerance limit (Seinhorst, 1998). The relative crop yield was calculated as the crop yield for a given *Pi/*mean crop yield at *Pi* = 0. Twenty data per treatment and cropping season were used. Seinhorst’s damage function models obtained per each crop were contrasted considering confidence intervals at 95% of *m* and *T*, and a general model was constructed with pooled data when no differences were found.

*Pi* were grouped in classes represented in both treatments in order to determine the effect of grafting (*Pi* < *T)* and nematode densities (*Pi* > *T)* on fruit quality. Data were submitted to non-parametrical analysis by the npar1way procedure to compare between grafted and ungrafted plants for a given *Pi* classes by the Wilcoxon test and by the Kruskal–Wallis test to determine the effect of nematode densities per treatment per each cropping season.

The number of nuclei and GC per feeding site, the volume of each GC and the volume of GC per feeding site from the histopathology study were compared between resistant and susceptible germplasm per each crop using the JMP v.15 (SAS Institute, Inc.) software. Data were submitted to non-parametric Wilcoxon test or Student’s *t*-test (*P* < 0.05).

## Results

### Damage Function Models

**FIGURE 1:**
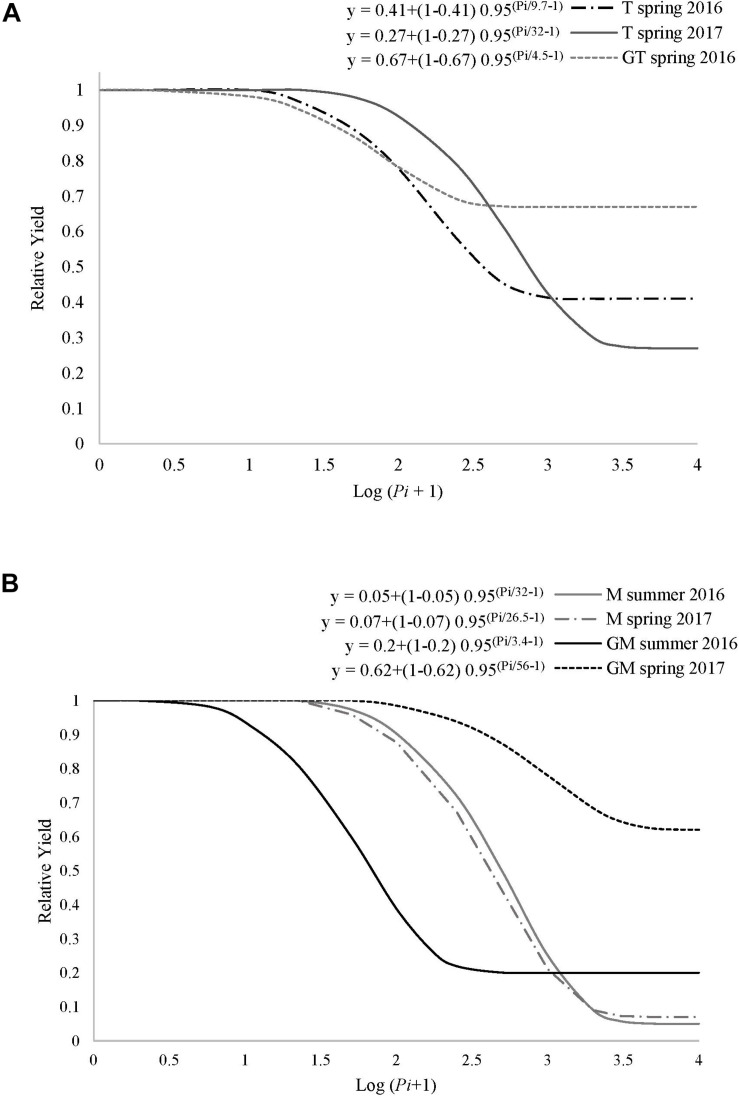
Seinhorst damage function model *y* = *m* + (1-*m*) 0.95 ^(Pi/T–1)^, where *y* is the relative crop yield, *m* is the minimum relative yield, *Pi* is the nematode population density at transplanting and *T* is the tolerance limit for **(A)** ungrafted tomato cv. Durinta (T) or grafted onto the resistant rootstock ‘Aligator’ (GT); and for **(B)** ungrafted melon cv. Paloma (M) or grafted onto the resistant rootstock *C. metuliferus* (GM) cultivated in *M. incognita* infested soil in a plastic greenhouse.

The relationship between *Pi* and the relative crop yield fitted the Seinhorst’s damage model for both ungrafted and grafted tomato and melon crops in 2016 and 2017 and some cropping seasons (Figures 1A,B). Minimum and maximum average soil temperatures at 15 cm depth during spring crops were 13.1 and 31.9 °C, respectively, and 17.1 and 30.6 °C during the summer crops. Grafted and ungrafted tomato cultivated in spring in non-infested plots yielded 4.1 and 3.9 kg plant^–1^ on average, respectively, and 2.2 and 2.0 kg plant^–1^ when cultivated in summer. At the end of the spring tomato crop cultivated in 2016, 4 out of 5 plots cultivated with ungrafted plants in non-infested soil were reinfested by the same nematode population. *Pi* densities at the beginning of the following melon crop ranged from 0 to 3494 J2 250 cm^–3^ of soil. In spring 2016, the minimum relative crop yield (*m*) and the tolerance (*T*) of grafted tomato cultivated in a *Pi* range from 0 to 1237 J2 250 cm^–3^ were 0.67 ± 0.03 and 5 ± 2 J2 250 cm^–3^ of soil, respectively (*R^2^* = 0.99, *P* < 0.05). For ungrafted tomato cultivated in a *Pi* range from 0 to 1496 J2 250 cm^–3^ of soil, the *T*-value (10 ± 7 J2 250 cm^–3^ of soil) did not differ from that estimated for the grafted one, but the *m-*value did (0.41 ± 0.19). In spring 2017, *m-* and *T-*values for ungrafted tomato cultivated in a *Pi* range from 0 to 2174 J2 250 cm^–3^ of soil were 0.27 ± 0.26 and 32 ± 25 J2 250 cm^–3^ of soil, respectively, (Figure 1A) and did not differ from those estimated in spring 2016. Then, a single model was constructed with the pooled data for ungrafted tomato, which provided estimated values of *m* and *T* of 0.34 ± 0.12 and 15 ± 7 J2 250 cm^–3^ of soil, respectively (*R^2^* = 0.96, *P* < 0.0001). The relationship between *Pi* and the relative tomato crop yield cultivated in summer did not fit the Seinhorst damage function model, irrespective of grafting.

Regarding melon, grafted and ungrafted melon cultivated in non-infested plots in spring yielded on average 2.5 and 2.4 kg plant^–1^, respectively, and 1.5 and 1.6 kg plant^–1^ when cultivated in summer. At the end of the spring melon crop cultivated in 2016, 4 out of 5 plots cultivated with ungrafted plants in non-infested soil were reinfested by the same nematode population. *Pi* in the following tomato crop ranged from 0 to 241 J2 250 cm^3^ of soil. Values of *m* and *T* for ungrafted crop cultivated in a *Pi* range from 0 to 7306 J2 250 cm^–3^ of soil in summer 2016 were 0.06 ± 0.06 and 32 ± 11, respectively (*R^2^* = 0.94; *P* < 0.0001) (Figure 1B). Concerning grafted melon cultivated in a *Pi* range from 0 to 12258 J2 250 cm^–3^ of soil in summer 2016, the estimated *m-* and *T-*values were 0.2 ± 0.08 and 3 ± 3 J2 250 cm^–3^ of soil, respectively (*R^2^* = 0.97; *P* < 0.0001). In spring 2017, *m-* and *T-*values for grafted melon were 0.62 ± 0.1 and 56 ± 32 J2 250 cm^–3^ of soil, respectively, when cultivated in a *Pi* range from 0 to 6086 J2 250 cm^–3^ of soil (*R^2^* = 0.99; *P* < 0.0001), and 0.07 ± 0.05 and 27 ± 6 J2 250 cm^–3^ of soil, respectively, for ungrafted melon cultivated in a *Pi* range from 0 to 6680 J2 250 cm^–3^ of soil (*R^2^* = 0.99; *P* < 0.0001). The estimated Seinhorst damage function models for ungrafted melon cropped in summer 2016 and in spring 2017 did not differ according to the confidence interval values of *m* and *T*. Consequently, a single model was constructed with the pooled data for ungrafted melon. The estimated *m-* and *T*-values were 0.02 ± 0.02 and 33 ± 7 J2 250 cm^–3^ of soil, respectively (*R^2^* = 0.97; *P* < 0.0001).

### Fruit Quality

The range (minimum and maximum values) of the fruit quality parameters of tomato and melon fruits produced on ungrafted and grafted plants cultivated in spring or summer in infested and non-infested soil are presented in Tables 1, 2, respectively.

**TABLE 1:** Values of fruit quality parameters (minimum and maximum) of the tomato cv. Durinta ungrafted (T) and grafted (GT) onto cv. Aligator, cultivated in infested and non-infested *M. incognita* plots in plastic greenhouse in spring or summer during 3 years (2015–2017), and those reported by the department of Agriculture of United States of America (USDA), and by Coyago-Cruz et al. (2017) for the cluster tomato cv. Tigerella, Palamós and Byelsa, and the cherry tomato cv. Lazarino and Summerbrix.

Parameter	GT				T				USDA*	Coyago-Cruz et al., 2017
	Spring		Summer		Spring		Summer			
	Infested	Non-infested	Infested	Non-infested	Infested	Non-infested	Infested	Non-infested		
L*	38.2 – 42.6	38 – 44.7	40.3 – 44.6	43 – 46.3	37.5 – 40.5	37.8 – 41.2	38 – 47.1	40.4 – 41.1	n.a	33.4 – 43.9
AE	31 – 41.7	30.2 – 41.8	39.4 – 44.4	41.1 – 47	30 – 43.9	32.1 – 42.7	39.9 – 47.8	41.3 – 43.7	n.a	n.a
Chroma	26.6 – 38.2	19.3 – 41.5	36 – 41.5	35.8 – 39.3	21.8 – 39.6	21.5 – 41	34.6 – 44.6	38 – 40.3	n.a	31.6 – 46.7
Hue	37.4 – 52.8	37.7 – 63.3	37.5 – 45.6	38.8 – 49	38.2 – 48.1	37.2 – 51.8	37.3 – 49.5	38.1 – 39	n.a	40 – 63.7
*TSS* (°Brix)	3.5 – 5.8	3.5 – 5.3	4.3 – 5.4	4.6 – 5.4	4 – 6.6	3.9 – 5.8	4.1 – 5.1	4.5 – 4.7	n.a	4.7 – 7.9
*dm* (%)	5.9 – 7.3	6 – 8.5	5.7 – 6.7	6 – 7	5.8 – 8.5	6.4 – 7.1	5.6 – 6.8	5.9 – 6	5.5	n.a
*Lycopene* (mg lycopene kg *dw*^–^*^1^*)	251 – 885	37 – 1275	332 – 457	371 – 485	90 – 976	113 – 1185	261 – 704	261 – 390	396	252 – 1510
*T.A* (g citric acid kg *dw*^–1^)	6.6 – 7	4.7 – 7	6.1 – 8.9	7 – 7.5	0.2 – 7.4	0.2 – 6.9	0.4 – 0.9	0.4 – 0.8	n.a	n.a
*TPC* (g GAE kg *dw*^–1^)	4.1 – 7	1.8 – 5.3	4.2 – 6	4.3 – 7	3.6 – 6.1	3.8 – 4.8	3.1 – 6.2	3.5 – 7.4	n.a	2.2 – 4.3
*Vitamin C* (g ascorbic acid kg *dw*^–1^)	1.5 – 4.1	1.5 – 6.7	2.3 – 2.7	2.4 – 3.6	1.9 – 3.6	1.5 – 3.1	1.8 – 3.4	3 – 3.6	2.1	n.a
*Antioxidant activity* (mmol Trolox kg *dw*^–1^)	8.2 – 57	8.4 – 63.8	28 – 81.2	26.8 – 66.3	8.7 – 79.6	10.7 – 74.2	20.3 – 70.4	41.4 – 57.5	n.a	n.a
*pH*	4 – 4.5	3.9 – 4.5	3.9 – 4.4	4 – 4.4	3.9 – 4.5	4 – 4.5	3.9 – 4.4	4.2 – 4.3	n.a	n.a
*mm* (%)	7.9 – 12.2	6.8 – 9	8 – 8.9	7.8 – 9.4	7.7 – 9.8	7.6 – 9.2	7 – 9	8.3 – 9.1	n.a	n.a
*Fe* (mg kg *dw*^–1^)	43.6 – 75.3	34.7 – 73.2	46.5 – 66	50.1 – 68	11 – 99.2	31.4 – 85.3	48.6 – 66.1	53.3 – 69.7	41.5	n.a
*Ca* (g kg *dw*^–1^)	1.2 – 2.8	1.7 – 3.5	0.9 – 2.8	1.6 – 3.3	0.8 – 2.3	1.4 – 2.4	0.9 – 3.1	1.6 – 2.5	1.5	n.a
*Mg* (g kg *dw*^–1^)	1.1 – 1.6	1 – 1.7	1.2 – 1.5	1.3 – 1.5	1.1 – 1.7	1.2 – 1.7	1 – 1.6	1.4 – 1.6	1.7	n.a
*K* (g kg *dw*^–1^)	15.3 – 26.5	13.2 – 27	22.1 – 29	21.6 – 28.5	11.2 – 26.5	12.3 – 29.1	19.9 – 29.2	23.5 – 27.8	36.5	n.a
*Na* (g kg *dw*^–1^)	1.7 – 5	1.8 – 3.9	2 – 5	1.9 – 2.9	2 – 6.8	1.6 – 4	2.3 – 3.7	2.3 – 3.1	0.77	n.a

*The original values reported by U.S. Department of Agriculture [USDA] (2020a), and Coyago-Cruz et al. (2017) were adapted to the units used in this study n.a: Data not available.*

**TABLE 2:** Values of fruit quality parameters (minimum and maximum) of the cantaloupe melon cv. Paloma ungrafted (M) and grafted (GM) onto *C. metuliferus* BGV11135 cultivated in infested and non-infested *M. incognita* plots in plastic greenhouse in spring or summer during 3 years (2015–2017), and those reported by the department of Agriculture of United States of America (USDA), and by Colla et al. (2006) for the melon cantaloupe cv. Cyrano, grafted or ungrafted onto *C. maxima* x *C. moschata*, and by Lester (2008) for the honeydew melon cv. Orange Dew.

Parameter	GM				M				USDA*	Colla et al., 2006	Lester, 2008
	Spring		Summer		Spring		Summer				
	Infested	Non-infested	Infested	Non-infested	Infested	Non-infested	Infested	Non-infested			
L*	50.5 – 84.7	45.8 – 80	49.5 – 72.4	54.5 – 66.5	39.9 – 71.3	49.3 – 69.6	57.5 – 72.5	54.9 – 66.5	n.a	53.1 – 58.3	n.a
AE	51.6 – 68.9	50.6 – 59.7	49.2 – 58.4	50.5 – 54.6	53 – 59.7	50.6 – 55.3	50.5 – 56.2	58.4 – 61.4	n.a	n.a	n.a
Chroma	18.2 – 47.2	21.2 – 45.2	19.5 – 41.2	33 – 47.3	19.6 – 45.2	28.4 – 42.3	22.9 – 41.6	38.1 – 45.2	n.a	n.a	n.a
Hue	39.4 – 79.6	44.6 – 84.1	66.6 – 81.8	64.8 – 75.1	79.6 – 83.9	80.4 – 85.9	60.4 – 81.3	67.7 – 74.1	n.a	n.a	n.a
*TSS* (°Brix)	10.4 – 15.7	8.5 – 14.8	10 – 17	12.2 – 16	6.4 – 14.7	10.1 – 14.8	9 – 14.9	11.1 – 17.2	n.a	10.1 – 12.6	8.6 – 13.3
*dm* (%)	12.8 – 24.3	9.4 – 15.4	9.6 – 15.6	12.8 – 15.4	8.5 – 14.7	8.2 – 14	6.9 – 15.4	11.5 – 16.8	9.85	10.4 – 13.2	9 – 12.1
B-carotene (mg β-carotene kg *dw*^–^*^1^*)	18–157	12–136	20 – 102	38.3 – 51.7	14 – 151	13 – 73	19 – 54.3	31 – 40	206	n.a	214 – 215
*T.A* (g citric acid kg *dw*^–1^)	5.2 – 20.9	6.8 – 15.9	6.7 – 24.9	5.1 – 22.1	5.1 – 17.6	9.6 – 38.2	7.2 – 22.4	15 – 18	n.a	n.a	n.a
*TPC* (g GAE kg *dw*^–1^)	1 – 3.8	2 – 3.5	1.8 – 4.1	2.6 – 3.3	0.9 – 5.9	1.6 – 6	1 – 4	1.5 – 3.2	n.a	n.a	n.a
Vitamin C (g ascorbic acid kg *dw*^–1^)	0.8 – 2.4	0.8 – 1.9	1.4 – 2.2	1.4 – 1.9	1.1 – 2.3	0.4 – 1.6	1.1 – 2.5	1.3 – 1.7	3.7	n.a	1.3 – 1.4
Antioxidant activity (mmol Trolox kg *dw*^–1^)	20.3 – 42.3	21 – 43.1	3.3 – 30.2	7.3 – 30	10.7 – 38.7	8.7 – 35.2	7 – 28.5	17.4 – 22.6	n.a	n.a	n.a
pH	4.9 – 6.9	5.6 – 7	5.1 – 6.7	6.1 – 6.5	5.6 – 7.6	6 – 6.9	5.1 – 6.2	5.7 – 6.5	n.a	6 – 6.7	n.a
*mm* (%)	6 – 14.2	7.8 – 12.3	7.8 – 12.2	6.6 – 11.6	6.4 – 11.6	7 – 14	6.2 – 8.6	6.5 – 8.9	n.a	n.a	n.a
Fe (mg kg *dw*^–1^)	27 – 70	41.9 – 70	49 – 60.6	54.7 – 61	32 – 85	43.5 – 81.7	45.2 – 63	43.1 – 53.7	21.4	n.a	20
Ca (g kg *dw*^–1^)	1.4 – 2.5	1.3 – 2	0.8 – 3.2	0.9 – 2.6	1.6 – 2.2	1.7 – 2.4	1.4 – 3.4	1.6 – 2.6	0.9	n.a	0.1
Mg (g kg *dw*^–1^)	0.7 – 1.3	0.9 – 1.2	0.8 – 2.1	0.9 – 2.1	0.9 – 1.7	1 – 1.7	0.9 – 2.3	1 – 1.9	1.2	n.a	0.6 – 0.9
K (g kg *dw*^–1^)	17.4 – 24.1	19.5 – 25.9	15.3 – 23.9	17.3 – 21.1	23.6 – 24.8	21.9 – 27	19.4 – 21.9	18.1 – 20	27.2	31.3 – 34.9	21.6 – 23.2
Na (g kg *dw*^–1^)	2 – 3.4	2.1 – 3.3	2.3 – 4.6	2.3 – 4.6	2.7 – 13.4	3 – 4.2	3 – 6.2	3.9 – 4.7	1.6	0.9 – 14	1.6 – 2.6

*The original values reported by U.S. Department of Agriculture [USDA] (2020b), Colla et al. (2006) and Lester (2008) were adapted to the units used in this study n.a: Data not available.*

**FIGURE 2:**
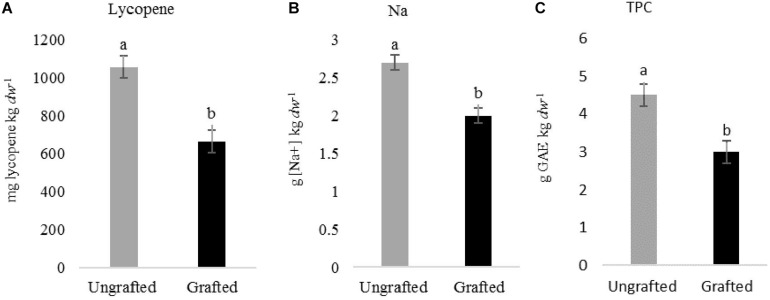
Effect of grafting on lycopene **(A)**, sodium concentration [Na^+^] **(B)** and total phenolic compound **(C)** in tomato cv. Durinta fruits produced in spring 2016. Data are mean ± standard error (*n* = 5). Column with the same letter did not differ (*P* < 0.05) according to the non-parametric Wilcoxon test.

**FIGURE 3:**
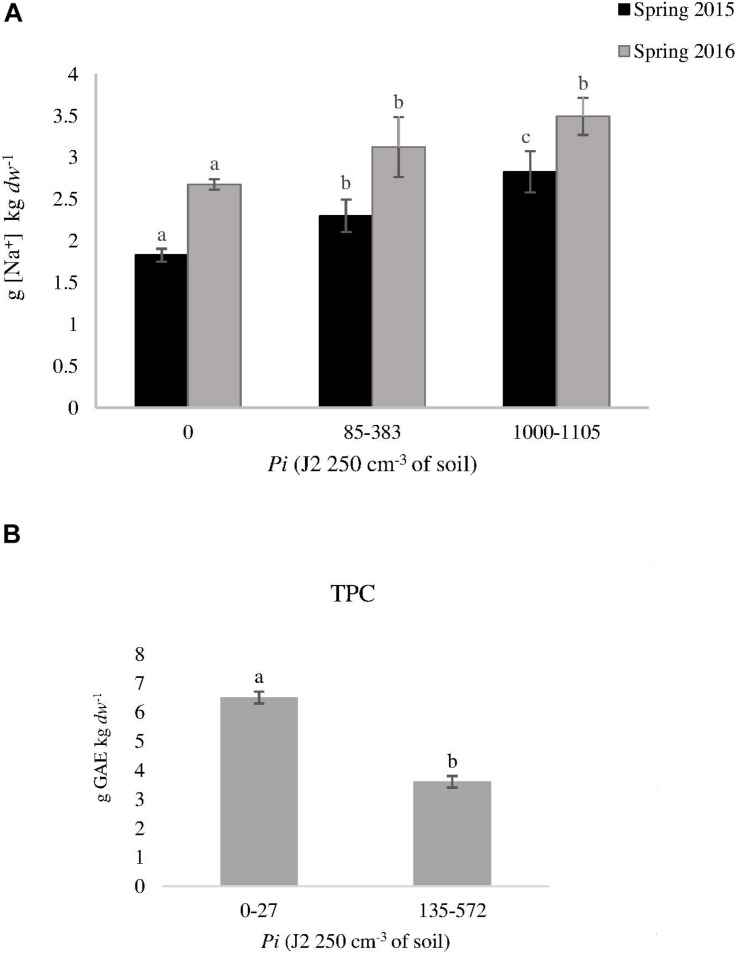
Effect of nematode density at transplanting (*Pi*) on **(A)** sodium concentration [Na^+^] in tomato fruits produced on ungrafted tomato cv. Durinta (T) cultivated in spring 2015 and 2016, and on **(B)** phenolic compounds in summer 2016. Data are mean ± standard error (*n* = 5). Column of the same year with the same letter did not differ (*P* < 0.05) according to the non-parametric Kruskal–Wallis test **(A)** or to the non-parametric Wilcoxon test **(B)**.

Tomato fruit quality parameters produced on plants cultivated in spring and summer 2015 and in spring 2017 in non-infested plots did not differ (*P* > 0.05) irrespective of grafting. However in 2016, lycopene, Na and *TPC* were higher (*P* < 0.05) in fruits produced in ungrafted than in grafted plants (1057 ± 71 *vs.* 663 ± 45 mg lycopene kg^–1^ *dw*; 2.7 ± 0.1 *vs.* 2 ± 0.1 g of Na kg^–1^ *dw*; and 4.5 ± 0.2 *vs.* 3 ± 0.5 g *GAE* kg^–1^ *dw*) (Figures 2A–C). Increasing nematode densities did not affect (*P* > 0.05) any of the tomato fruit quality parameters from grafted plants, but it did from ungrafted ones. The Na concentration in tomato fruits produced on ungrafted plants cultivated in infested plots was higher than those cultivated in non-infested plots in spring 2015 and 2016 (Figure 3A). Moreover, lower (*P* < 0.05) *TPC* was found in fruits from ungrafted tomato plants cultivated in a *Pi* range from 135 to 572 J2 250 cm^–3^ of soil (3.6 ± 0.1 g *GAE* kg^–1^ *dw*) than those cultivated in a *Pi* range from 0 to 27 J2 250cm^–3^ of soil (6.5 ± 0.3 g *GAE* kg^–1^ *dw*) in summer 2016 (Figure 3B).

Concerning melon, higher (*P* < 0.05) Na content was found in fruits from ungrafted plants respect to the grafted ones cultivated in non-RKN infested plots irrespective of the cropping season. Dry matter and *SSC* also differed (*P* < 0.05) between melon fruits produced on ungrafted and grafted plants cultivated in summer 2015 and spring 2016 (Table 3). However, higher (*P* < 0.05) Na and dry matter content were found in fruits produced on ungrafted melon cultivated in infested soil in spring 2015, as well as of Na and *SSC* when cultivated in spring 2017. About fruits produced on grafted plants, the majority of the quality parameters were not affected by RKN densities, except dry matter and *SSC* that were lower (*P* < 0.05) at high nematode densities when cultivated in summer but not in spring (Table 3).

**TABLE 3:** Soluble solid content (*SSC*), dry matter (*Dm*) and sodium content in ungrafted melon cv. Paloma (M) or grafted onto the resistant rootstock *C. metuliferus* BGV11135 (GM) cultivated in soil infested with increasing *Meloidogyne incognita* densities at transplanting (*Pi*) in a plastic greenhouse during 3 years (2015–2017).

Year	Season	*Pi* range (J2 250cm^–3^)	*SSC* (°Brix)		*Dm* (%)		Na (g kg^–1^ *dw*)	
			GM	M	GM	M	GM	M
2015	Spring	0	12.2 ± 0.3 a	12.2 ± 0.2 a	12.3 ± 0.2 a	12.5 ± 0.1 a	3.7 ± 0.2 a	4.9 ± 0.1 b*
		72–349	12.3 ± 0.3 a	10.0 ± 0.3 b*	12.4 ± 0.1 a	9.9 ± 0.2 b*	4.4 ± 0.2 a	8.5 ± 0.6 a*
		502–709	12.2 ± 0.4 a	10.5 ± 0.7 ab	12.3 ± 0.4 a	10.0 ± 0.3 b*	4.1 ± 1 a	8.3 ± 0.8 a*
	Summer	0	15.2 ± 0.2 a	16.4 ± 0.4*	14 ± 0.4 a	15.6 ± 0.4*	4.5 ± 0.4 a	6.1 ± 0.3*
		96–427	13.4 ± 0.5 b	n.a	12.3 ± 0.3 b	n.a	6.5 ± 0.8 a	n.a
2016	Spring	0	13.8 ± 0.4 a	12.6 ± 0.3 a*	14.0 ± 0.4 a	12.3 ± 0.3 a*	2.5 ± 0.1 a	3.6 ± 0.2 a*
		15–48	12.9 ± 0.1 a	12.8 ± 0.1 a	12.6 ± 0.4 a	12.4 ± 0.2 a	2.5 ± 0.1 a	3.9 ± 0.2 a*
	Summer	0^†^	13.3 ± 0.5 a	12.6 ± 0.7	14.1 ± 0.5 a	13.4 ± 0.8	3.1 ± 0.1 b	4.3 ± 0.4*
		1581–3772	10.8 ± 0.3 b	n.a	10.5 ± 0.9 b	n.a	3.8 ± 0.2 a	n.a
2017	Spring	0^†^	14.0 ± 0.2 a	14.7 ± 0.2 a	13.6 ± 0.7 a	13.7 ± 0.6 a	2.7 ± 0.2 a	4.1 ± 0.4 ab*
		203–951	13.1 ± 0.6 a	14.2 ± 0.2 a	12.5 ± 1.3 a	13.6 ± 0.5 a	1.8 ± 0.3 a	3.8 ± 0.2 b*
		1156–3476	11.9 ± 0.9 a	11.7 ± 0.5 b	12.8 ± 0.3 a	12.3 ± 0.5 a	2.3 ± 0.4 a	5.1 ± 0.3 a*

Data are mean ± standard error of 5 replicates. Data in the same column and cropping season followed by the same letter did not differ (*P* < 0.05) according to the non-parametric Mann–Whitney or Kruskal–Wallis test. Data within the same row per quality parameter followed by * indicate differences between germplasm according to the non-parametric Mann–Whitney test (*P* < 0.05). n.a: Range of *Pi* not represented in the treatment; ^†^: *Pi* = 0 included nematode densities below the plant tolerance according to the Seinhorst damage function model.

### Optical Histopathology

**FIGURE 4:**
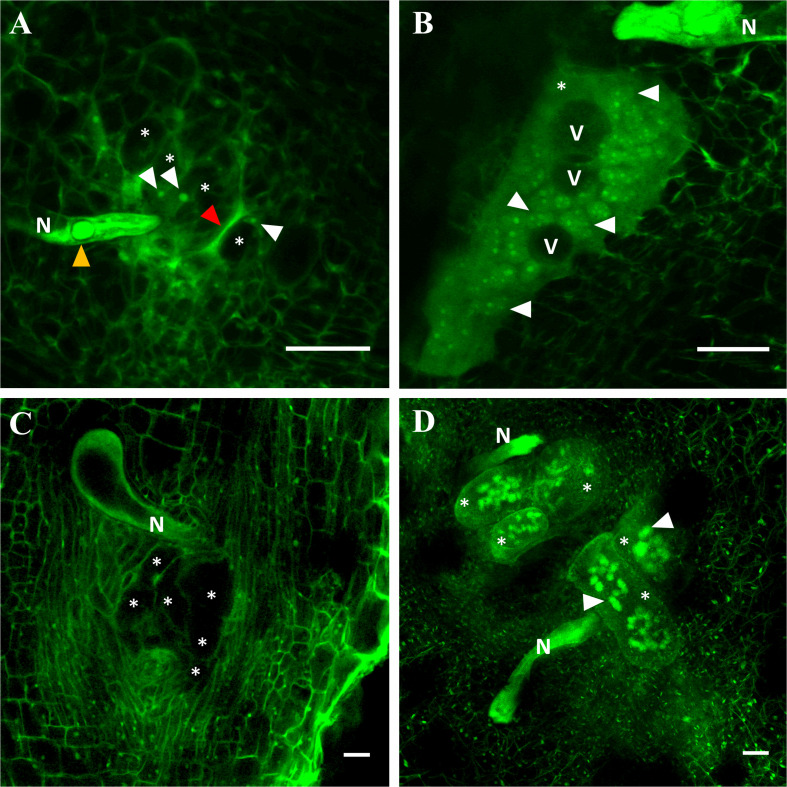
Laser scanning confocal microscope images of giant cells induced by *Meloidogyne* 15 days after inoculation in the resistant *Cucumis metuliferus* BGV11135 **(A)**, the susceptible melon cv. Paloma **(B)**, the resistant tomato cv. Monika **(C)** and the susceptible cv. Durinta **(D)**. Nematode (N), vacuoles (V), giant cells (asterisk), some nuclei (white arrowhead), esophageal median bulb (yellow arrowhead) and necrosed area (red arrowhead) are indicated. Scale bar: 50 μm.

Fifteen days after *M. incognita* inoculation, the nematode induced 1.8 more (*P* < 0.05) giant cells (GCs) in *C. metuliferus* than in melon cv. Paloma, but they were less (*P* < 0.05) voluminous (94.3%) holding 92.9% fewer (*P* < 0.05) nuclei per GC. Both GCs volume and number of nuclei per feeding site were higher (*P* < 0.05) in susceptible melon than in *C. metuliferus* (Table 4). Some GCs in *C. metuliferus* did not emit fluorescence and no nuclei were observed compared to those observed in the susceptible melon cv. Paloma which were more voluminous, multinucleated and vacuolated (Figures 4A,B, Supplementary Figure S1 and Supplementary Videos S1, S2).

**TABLE 4:** Giant cell volume (GCV), GC volume per feeding site (GCV fs**^–^**^1^), number of nuclei per GC (N GC**^–^**^1^), number of nuclei per feeding site (N fs**^–^**^1^), and number of cells per feeding site (NC fs**^–^**^1^) in the resistant (R) *C. metuliferus* BGV11135 and tomato cv. Monika and the susceptible (S) melon cv. Paloma and tomato cv. Durinta 15 days after nematode inoculation with 3 or 1 J2 cm**^–^**^3^ of soil, respectively, and cultivated in 200 cm^3^ pots in a growth chamber.

Host plant (host status)	GCV (μm^3^ 10^5^)	GCV fs^–1^ (μm^3^ 10^5^)	N GC^–1^	N fs^–1^	NC fs^–1^
*C. metuliferus* (R)	0.45 ± 0.1*	3.41 ± 0.8*	1.2 ± 0.7*	9.2 ± 5.5*	8.0 ± 1.1*
Melon cv. Paloma (S)	7.97 ± 1.5	33.19 ± 9.9	17.1 ± 1.8	72.0 ± 7.8	4.5 ± 1.0
Tomato cv. Monika (R)	3.14 ± 0.4*	26.84 ± 3.7	0.9 ± 0.4*	7.0 ± 3.0*	8.7 ± 1.2*
Tomato cv. Durinta (S)	11.42 ± 1.9	45.94 ± 7.3	13.7 ± 1.0	56.2 ± 7.3	4.1 ± 0.4

Data are the mean ± standard error of 4 replications. Data in the same column followed by * indicates differences (*P* < 0.05) between *Cucumis* species or tomato cultivars according to the non-parametric Wilcoxon test or Student’s *t*-test.

Regarding tomato, 2.1 more (*P* < 0.05) GCs were induced in the resistant tomato cv. Monika than in the susceptible cv. Durinta, but they were 72.5% less (*P* < 0.05) voluminous and had 93.3% fewer (*P* < 0.05) nuclei per GC (Table 4). However, GCs volume per feeding site did not differ between tomato cultivars, but the number of nuclei per feeding site did, being higher (*P* < 0.05) in susceptible than in resistant tomato (Table 4). In resistant tomato, several GCs did not emit fluorescence and no nuclei were observed compared to the voluminous and multinucleated GCs observed in the susceptible tomato (Figures 4C,D, Supplementary Figure S1 and Supplementary Video S1).

## Discussion

The results of this study provide novel information on the effect of nematode densities and the cropping season on grafted tomato and melon tolerance to *M. incognita*, crop yield losses, and fruit quality.

Expósito et al. (2019) found that tomato yield did not differ between ungrafted and grafted tomato onto the tomato rootstock ‘Aligator’ cultivated in non-nematode infested soil, but it did in infested. The results of the present study have shown that the tolerance of ungrafted and grafted tomato cv. Durinta onto ‘Aligator’ to *M. incognita* cultivated in the same season and year did not differ but the later suffered a 36% less relative yield losses (59% *vs.* 23%). Di Vito et al. (1991) found that the tolerance to *M. incognita* of the susceptible cv. Ventura and the resistant cv. Disa N did not differ (0.55 J2 cm^–3^ of soil) but yield losses were lower in the resistant than in the susceptible tomato (30% *v*s 100%) in microplot conditions. In our study, the tolerance to *M. incognita* of the susceptible tomato cv. Durinta cultivated in spring was similar to that previously reported by Giné and Sorribas (2017).

Grafting did not influence the majority of fruit quality parameters of tomato cultivated in non-infested soil, except lycopene, Na and *TPC* that were lower in fruits from grafted than ungrafted plants but only in one out of 3 years. It is known that grafting can affect tomato fruit quality depending on the scion-rootstock combination and environmental conditions, including abiotic and biotic factors (Fernández-García et al., 2004; Turhan et al., 2011; Vrcek et al., 2011; Di Gioia et al., 2013; Erba et al., 2013). Nonetheless, Grieneisen et al. (2018) conducted an extensive review of data from 159 publications to point light on the effect of grafting on tomato yield and fruit quality. They concluded that grafting rarely causes fruit quality changes and that self-grafted plants yielded similarly than ungrafted plants. However, the occurrence of abiotic and/or biotic stresses and its intensity during a given phenological stage of the plant can lead to changes in fruits and vegetables quality such as an increase of bioactive compounds (Nicoletto et al., 2019; Toscano et al., 2019). Interestingly, there is a crossing-talk between signaling pathways allowing plant plasticity to be adapted to environmental situations (Martinez-Medina et al., 2017; Ghahremani et al., 2020). Atkinson et al. (2011) studied the effect of water stress and *M. incognita* (10 eggs g^–1^ soil) alone and in combination on the nutritional fruit quality of tomato cv. Shirley cultivated in pots in a growth chamber. They found that the second cluster produced by nematode inoculated plants had less dry matter content than that produced by non-inoculated, contrarily to the results obtained from the fifth cluster that in addition had more content of phenolic compounds. When both kinds of stresses were combined, the percentage of fruit dry matter of the second cluster was similar to that the water stressed plants alone. It seems that the initial nematode densities at transplanting was not enough to affect the quality of the second cluster fruits but increasing nematode density after completion of the first generation affected the fifth cluster. In our study, that was conducted in non-controlled conditions, in which the third cluster fruit was used for assessing fruit quality parameters when they reached the commercial standards, increasing nematode densities at transplanting did not affect the quality of fruits produced by grafted plants. However, the *TPC* in fruits from ungrafted tomato decreased at nematode densities between 135 and 572 J2 250 cm^–3^ of soil in summer 2016, and Na concentration increased in spring 2015 and 2016. The range of Na content in tomato fruits were between 2.1 and 8.8 times higher than that reported by U.S. Department of Agriculture [USDA] (2020a) (Table 1). The tomato cultivar and crop management can affect the concentration of nutritional compounds as it has been reported by Erba et al. (2013) who found values of Na content in three tomato cultivars between 4.8 and 17.6 higher than that reported by U.S. Department of Agriculture [USDA] (2020a) depending on the tomato cultivar, N fertilization, and fungicide application.

In relation to melon, Expósito et al. (2019) found that the yield of ungrafted and grafted melon onto *C. metuliferus* cultivated in non-nematode infested soil did not differ irrespective of the cropping season. In the present study, the estimated tolerance to *M. incognita* of ungrafted and grafted melon cultivated in spring did not differ but maximum yield losses did, being 98% for ungrafted and 38% for grafted melon. Reports about grafted melon tolerance to RKN and yield losses are scarce. Kim and Ferris (2002) estimated the tolerance to *M. arenaria* and yield losses of melon cv. Geumssaragi-euncheon grafted onto the *Cucurbita* hybrid rootstock ‘Shintoza’ cultivated at nematode densities between 0 and 2980 J2 per 100 cm^–3^ of soil, being 0 J2 100 cm^–3^ of soil and 57%, respectively. According to these results, *C. metuliferus* is more tolerant to RKN and experience less yield losses than the *Cucurbita maxima* x *C. moschata* rootstock. In fact, plant tolerance and crop yield losses of grafted cucumber onto the *Cucurbita* hybrid rootstock ‘RS841’ did not differ from ungrafted but the nematode population growth rate did, being higher in grafted than ungrafted cucumber, indicating that it was not resistant to the nematode (Giné et al., 2017). Plant species supporting high nematode population growth rates leave high nematode densities at the end of the crop causing more yield losses to the following one. *C. metuliferus* has been proven to suppress nematode population growth rate compared to melon, being an indicator of its resistance against the nematode (Expósito et al., 2018). Under an agronomic point of view, rootstocks bearing resistance and tolerance genes to RKN are needed to manage them and to avoid crop yield losses.

Regarding melon fruit quality, it has been reported that the *C. metuliferus* accession BGV11135 did not affect physical fruit traits, *SSC* and pH when cultivated in hydroponic system (Expósito et al., 2018). But fruit quality can be affected according to the scion-rootstock combination and the cultivation system. For example, Guan et al. (2014) did not find differences on flesh firmness and *SSC* between ungrafted melon cv. Honey Yellow and grafted onto *C. metuliferus* cultivated under both conventional and organic standards, but did in fruits from grafted melon cv. Arava cultivated under both cropping systems as well as less *SSC* was found when cultivated under conventional system. In our study, lower Na content was measured in fruits from grafted than ungrafted plants cultivated in non-infested soil. Interestingly, increasing nematode densities increased Na content in fruits from ungrafted but not from grafted plants. Nonetheless, the levels of Na reached in melon fruits from both grafted and ungrafted plants (1.8 to 8.5 g Na kg^–1^ *dw*) were in the range of that reported by Colla et al. (2006) but slight higher in ungrafted melon than that reported by Lester (2008) and U.S. Department of Agriculture [USDA] (2020b) (Table 2). Furthermore, increasing nematode densities reduced the *SSC* and the dry matter content in fruits produced in ungrafted plants in spring and in those produced in grafted plants cultivated in summer. Ploeg and Phillips (2001), found an increase in the percentage of dry matter of the areal plant part of melon cv. Durango after 8 weeks of cultivation in pots non-inoculated and inoculated with an increasing nematode density from 0.06 to 15 J2 100 g^–1^ of soil. In field conditions, significant yield reduction was observed due to a reduction in the number of fruits at increasing nematode densities over *T.* It seems that the metabolic activity of the nematode would compete with fruit development which could be inhibited.

In this line, the effect of suboptimal growing conditions, as for example high temperatures and radiation levels which are achieved in the Mediterranean areas at transplanting during the summer season can affect plant metabolism. Heat stress can affect plant photosynthesis and the phenylpropanoid pathway. Moreover, ROS can be accumulated in the tissues and the plant will activate antioxidants mechanisms to protect cell structures from oxidation. In addition, light excess can induce severe damage to the photosystem II (Toscano et al., 2019). These stresses will lead to a reduction in the potential yield of the crop and potential changes in the fruit quality. Thus, the selection of the best season for cropping is also necessary to maximize its efficiency as it was previously described for cucumber-*M. incognita* and for zucchini-*M. incognita* (Giné et al., 2014, 2017; Vela et al., 2014). These studies found that cucumber and zucchini were more tolerant and suffered lower yield losses when cultivated in spring than in summer or autumn. Similar results were observed in our study for grafted melon, which was more tolerant and experienced less yield losses when cultivated in spring instead of summer. So, it is expected that the damage of the nematode infection increase and the tolerance were reduced under those stressful conditions due the required energy to overcome RKN infection and the abiotic stress together. Grafting onto tolerant rootstocks has been used widely to overcome the damage to different abiotic stresses, including high temperatures (Tao et al., 2020). Consequently, screening for resistant-RKN and tolerance to abiotic stress will increase the availability of scion-rootstock combinations for agriculture production to overcome RKN and sub-optimal growing conditions.

The histopathological study provided interesting information related to the number and volume of giant cells and the number of nuclei into them. Giant cells formation is a key factor for a successful plant-nematode interaction after the nematode arrive into the cortical cylinder. The induced multinucleated giant cells have a high metabolic activity necessary for nematode nutrition for its life cycle completion (Abad et al., 2009). Conversely, if giant cells are not formed or appear as degenerated holding none or few nuclei, the nematode development and/or reproduction will be suppressed indicating a resistant response of the plant. Cabrera et al. (2015) used 3D reconstructions of GCs induced by *M. javanica* in *Arabidopsis* roots, and to compare GCs formed in the *Arabidopsis* transgenic line J0121 > > DTA, in which the GCs are genetically ablated, with a control (line J0121 > > GFP). These authors found that the GCs volume in the control was 2 fold larger. The results of our study have shown that both resistant *C. metuliferus* and tomato cv. Monika had more number of giant cells per feeding site than melon and susceptible tomato 15 days after *M. incognita* inoculation, but they were smaller, less voluminous, with fewer nuclei and some of them were empty of cytoplasm. Previous histopathological studies reported some of the observations pointed out in this study. Fassuliotis (1970) observed small GCs in *C. metuliferus* accession C-701 compared with those induced by *M. incognita* in melon; the nematode developed slow and a 20% of juveniles’ differentiated to males. Walters et al. (2006) observed elongated GCs conforming abnormal in shape feeding sites in *C. metuliferus* accession 482454 compared with melon. More recently, Ye et al. (2017) observed that the most of the GC were empty of cytoplasm in the *C. metuliferus* accession PI 482443-*M. incognita* interaction 14 days after nematode inoculation along with a slow nematode development compared with melon. Expósito et al. (2018) reported poorly GC development with multiple vacuoles, some of them without cytoplasm and necrotic areas surrounding the nematode head in the *C. metuliferus* accession BGV11135–*M. javanica* interaction compared to cucumber. Interestingly, the major number of GCs found in both resistant *C. metuliferus* and tomato could be due to an attempt of the nematode to achieve enough nutrients for its life cycle completion. In fact, the development of small GCs holding low number of nuclei could indicate a low effective metabolic activity for nematode nourishment. This strategy to achieve nutrients can have a biological cost for the nematode resulting in a slow development rate, as it was previously reported for both *C. metuliferus* and *Mi1.2* resistant tomato as well as for other resistant germplasms (Fassuliotis, 1970; Pedrosa et al., 1996; Walters et al., 2006; Williamson and Roberts, 2009; Ye et al., 2017).

Our research pointed out the importance to use grafted fruiting vegetables onto resistant rootstocks to decrease yield losses caused by RKN without conferring significant non-desirable quality traits. According to our data, the use of grafted plants could not be necessary to increase crop yield in absence of RKN because crop yield did not differ in our scenario. Nonetheless, rootstocks also bear other sources of resistance against soil-borne plant pathogens increasing its interest to be included in integrated disease management strategies. For example, *C. metuliferus* is also resistant to Monosporascus root rot and Fusarium wilt as well as to vine decline (Castro et al., 2020). Some other putative hybrid *Cucumis* rootstocks, such as *C. ficifolius* x *C. anguria* and *C. ficifolius* x *C. myriocarpus*, which are tolerant to *Monosporascus cannonballus*, and resistant to *Fusarium oxysporum* f.sp *melonis* and to RKN and did not affect the quality of melon fruit compared to non-grafted or self-grafted (Cáceres et al., 2017), will increase the number of possible rootstocks that could be available for growers in the near future. Special attention should be pay to the selection of the optimal cropping season in order to maximize the performance of grafted plants as it was observed in this study. The main effect of RKN on tomato and melon yield was on quantity but not in quality since the most fruit quality parameters assessed were in the range of values previously reported for these crops.

## Data Availability Statement

The original contributions presented in the study are included in the article/Supplementary Material, further inquiries can be directed to the corresponding author.

## Author Contributions

FJS, AG, AE, and NE conceived and designed the experiments. FJS supervised the experiments, the data collection and analyses. AE performed the field experiments, analyzed the data and wrote the draft of the manuscript. IA and MP performed the quality fruit analyses. NE, AMF, MC, and PL-A performed the histopathological study. AE, AG, NE, AMF, and FJS wrote the final version of the manuscript. All authors edited and approved the final manuscript.

## Conflict of Interest

The authors declare that the research was conducted in the absence of any commercial or financial relationships that could be construed as a potential conflict of interest.

## Supplementary Material

Supplementary Figure 1 Selected planes of Z-stack images of laser-scanning confocal microscopy of giant cells induced by *Meloidogyne* 15 days after inoculation in the resistant *Cucumis metuliferus* BGV11135 (A), the susceptible melon cv. Paloma (B), the resistant tomato cv. Monika (C) and the susceptible cv. Durinta (D). Nematode (N), vacuoles (V), giant cells (asterisk), some nuclei (white arrowhead), esophageal median bulb (yellow arrowhead) and necrosed area (red arrowhead) are indicated. Scale bar: 50 μm.

Supplementary Video 1 *Z*-stack images of laser-scanning confocal microscopy images of giant cells induced by *Meloidogyne* 15 days after inoculation in the resistant *Cucumis metuliferus* BGV11135 (A), the susceptible melon cv. Paloma (B), the resistant tomato cv. Monika (C) and the susceptible cv. Durinta (D). Scale bar: 50 μm.

Supplementary Video 2 3D rendering of confocal Z-stack images of giant cells induced by *Meloidogyne* 15 days after inoculation in the susceptible melon cv. Paloma. Scale bar: 40 μm.
